# 

**Published:** 1876-01

**Authors:** 


### CONTENTS:

### ORIGINAL COMMUNICATIONS.

ARTICLE I.—The American Academy of Dental Surgery. Proceedings of Special Meetings, October 19 and 20, 1875 ........ 385
ARTICLE II.—The Dental Pulp, its Pathology and Treatment. By J. B. Willmott, D. D. S., L. D. S ........ 395

### SELECTED ARTICLES.

ARTICLE III.—Celluloid ........ 404

ARTICLE IV.—American Dental Association ........ 407

### EDITORIAL, ETC.

Does the use of Celluloid Infringe the Cumming's Patent? ........ 425
A Strange Freak ........ 427
Dying on the Operating Table ........ 427

### BIBLIOGRAPHICAL.

Transactions of the American Medical Association for 1875 ........ 429
Vernon Galbray, or the Empiric ........ 429
Questions on the Structure and Development of the Human Teeth ........ 429
The American Agriculturist ........ 430
Vick's Floral Guide for 1876 ........ 430
Circulars of Information of the Bureau of Education ........ 430
Dentists' Appointment Book and Pocket Record ........ 431
The Physician's Diary for 1876 ........ 431
The Sanitarian ........ 431
The Herald of Health ........ 432

### MONTHLY SUMMARY.

Croton Chloral Hydrate in Neuralgia ........ 432

